# Phenotypic convergence of bacterial adaption to sub-lethal antibiotic treatment

**DOI:** 10.3389/fcimb.2022.913415

**Published:** 2022-11-17

**Authors:** Gui Nam Wee, Eun Sun Lyou, Jin-Kyung Hong, Jee Hyun No, Soo Bin Kim, Tae Kwon Lee

**Affiliations:** Department of Environmental and Energy Engineering, Yonsei University, Wonju, South Korea

**Keywords:** antibiotics, phenotype, bacterial adaptation, raman spectroscopy, flow cytometry

## Abstract

Microorganisms can adapt quickly to changes in their environment, leading to various phenotypes. The dynamic for phenotypic plasticity caused by environmental variations has not yet been fully investigated. In this study, we analyzed the time-series of phenotypic changes in *Staphylococcus* cells during adaptive process to antibiotics stresses using flow cytometry and Raman spectroscopy. The nine antibiotics with four different mode of actions were treated in bacterial cells at a sub-lethal concentration to give adaptable stress. Although the growth rate initially varied depending on the type of antibiotic, most samples reached the maximum growth comparable to the control through the short-term adaptation after 24 h. The phenotypic diversity, which showed remarkable changes depending on antibiotic treatment, converged identical to the control over time. In addition, the phenotype with cellular biomolecules converted into a bacterial cell that enhance tolerance to antibiotic stress with increases in cytochrome and lipid. Our findings demonstrated that the convergence into the phenotypes that enhance antibiotic tolerance in a short period when treated with sub-lethal concentrations, and highlight the feasibility of phenotypic approaches in the advanced antibiotic treatment.

## Introduction

Microorganisms can adapt rapidly to changes in their environment, resulting in a variety of phenotypes. Such emergence of phenotypic diversity is considered a result of gene expression changes in response to the environment. However, it remains unclear whether the phenotype continues to change in response to the adaptable abiotic stress or whether the phenotype converges at a certain time point (Jervis, 2006). Transcriptome and metabolome analysis by Horinouchi and his colleagues suggested that phenotypic diversity converges during adaptive evolution of *Escherichia coli* to ethanol stress (Horinouchi et al., 2015). Such constraint of phenotype in the adaptation process is a ubiquitous phenomenon that also appears in the evolution of plants and animals (Rosenblum et al., 2010; Xu et al., 2020). Previous studies have observed phenotypic convergences from the perspective of long-term evolution where they focused on the changes in phenotype as an outcome of genetic evolution. Since environmental changes may occur in the form of temporary disturbance, it is also necessary to pay attention on the plasticity of phenotype without genetic alteration even in a short-term.

The emergence of antibiotic resistance bacteria is one of the world’s most urgent public health problems. The prudent clinical and non-clinical use of antibiotics may slow the spread and emergence of new antibiotic resistant bacteria, but the threat will remain due to the increased range of antibiotic resistance and the rapid evolution of bacteria (Bernier and Surette, 2013). Antibiotic treatment is strongly associated with frameshift mutations resulting in multidrug resistance (Pérez-Capilla et al., 2005). Antibiotics resistance has been considered to occur in antibiotics that require high therapeutic levels, but there is evidence that low-level antibiotic treatment can also lead to mutation that cause resistance (Girgis et al., 2009). Concentrations below the minimal inhibitory concentrations (MICs) of certain antibiotics are present in the human body during antibiotic therapy and these sub-lethal concentrations can also be found in many natural environments, such as sewage water and sludge, rivers, and lakes (Hermsen et al., 2012; Andersson and Hughes, 2014). Such sub-lethal levels of antibiotics act as a stress inducer, allowing rapid bacterial adaptation through a variety of biological responses by the bacteria, such as gene expression as well as phenotypic changes without genetic alternation (Andersson and Hughes, 2014). Although studies on the convergence of phenotypes through genotype-phenotype mapping through re-sequencing have been performed to understand long term adaptive evolution, qualitative understanding of the phenotypic adaptation remains unclear (Andersson and Hughes, 2014; Suzuki et al., 2014). To address this issue, a physiological approach at high resolution is needed to monitor phenotypic changes at sub-lethal concentrations of antibiotic treatment.

Optic-based technologies, including Raman spectroscopy and flow cytometry, can offer new insight into cellular phenotype at the single cell level and further our understanding about how microorganisms respond to abiotic stress (Garcia-Timermans et al., 2020; Hatzenpichler et al., 2020). Raman spectroscopy has provided useful biomolecule information as fingerprints of single cells, including lipid, carbohydrate, nucleic acid, and protein composition (Garcia-Timermans et al., 2020; Hong et al., 2021). Many studies have recently been conducted to identify antibiotic-resistant and sensitive strains using Raman spectroscopy and predict antibiotic mechanisms. These allows to develop the rapid assay of the minimal inhibitory concentration of antibiotics and determine the multi-resistant clinical strains in hospital (Kirchhoff et al., 2018; Barzan et al., 2020; Nakar et al., 2022). Furthermore, the extent of cellular damage and resulting Raman spectral changes have been found to play an important role in distinguishing antibiotic exposure characteristics. This indicates that Raman spectroscopy has the potential for rapid bacterial identification and antibiotic susceptibility profiling (Schröder et al., 2015), and may also be suitable for investigating phenotypic changes caused by antibiotic treatment (Germond et al., 2018).

In addition, flow cytometry (FCM) allows high throughput analysis of phenotypic heterogeneity at the single cell level. Furthermore, FCM is a simple and sensitive technique and is not only provides information on the total cell count of suspended cells, including cell viability status, but also provides information regarding phenotype (Müller and Nebe-Von-Caron, 2010). These physiological approaches are suitable for investigating phenotypic changes during rapid adaptation of microorganisms in response to abiotic stresses.

In this study, we analyzed phenotypic changes of the opportunistic pathogen *Staphylococcus aureus* under antibiotic treatment with sub-lethal concentrations. I treated *S. aureus* with nine different antibiotics at concentrations at the MIC and monitored the time-series of their phenotypic changes using Raman spectroscopy and FCM. Then, we compared the phenotypic diversity and biomolecular information of single cells to analyze the phenotype convergence in bacterial adaptation after short-term cultivation under antibiotic stress conditions.

## Material and methods

### Bacteria growth conditions and antibiotic treatments

*S. aureus* NCTC 8325-4 was grown in tryptic soy broth (TSB; BD, NJ, USA) at 37°C with shaking (120 rpm). Amoxicillin (AMX; Sigma-Aldrich, MO, USA), vancomycin (VAN; Sigma-Aldrich, MO, USA), gentamycin (GEN; Sigma-Aldrich, MO, USA), chloramphenicol (CHL; TCI, Tokyo, Japan), tetracycline (TET; Sigma-Aldrich, MO, USA), ciprofloxacin (CIP; Sigma-Aldrich, MO, USA), norfloxacin (NOR; Sigma-Aldrich, MO, USA), and rifampicin (RIF; TCI, Tokyo, Japan) were used as the antibiotics in this study. *S. aureus* was treated with these antibiotics at MICs. The modes of action of these antibiotics include inhibition of cell wall synthesis, protein synthesis, and DNA gyrase or RNA synthesis inhibition. The detailed information of the antibiotics and their MICs are summarized in **Table 1**. Optical density (OD) measurements of bacterial cultures were performed in a SPARK 10M microplate reader (TECAN, Männedorf, Switzerland) in a 96-well plate (SPL, Seoul, South Korea), with 200 μL per well. Absorbance was measured at a wavelength of 600 nm and the average value was obtained for triplicate measurements. I sampled the bacterial cultures at 6, 12, and 24 h for measuring single-cell phenotypes using Raman spectroscopy and FCM.

**Table 1:** Information of antibiotics and MIC of *Staphylococcus aureus* NCTC 8325-4.

Antibiotics	Class	Mode of action	Target	MIC (mg/L)	Reference
Amoxicillin (AMX)	Beta-lactam	Cell wall synthesis (CW)	Penicillin binding proteins (PBP)	0.06	(Stubbings et al., 2004)
Vancomycin (VAN)	Glycopeptides and glycolipopeptides	Cell wall synthesis (CW)	D-Ala-D-Ala moiety of NAM/NAG peptide subunits	1.25	(Hiramatsu et al., 1997)
Gentamycin (GEN)	Aminoglycosides	Protein synthesis Inhibitors (PS)	30S ribosomal protein S12 16S rRNA	0.25	(Stubbings et al., 2004)
Chloramphenicol (CHL)	Chloramphenicol	Protein synthesis Inhibitors (PS)	50S subunit of the ribosome and prevents the formation of peptide bonds	2	(Stubbings et al., 2004)
Tetracycline (TET)	Tetracycline	Protein synthesis Inhibitors (PS)	binding of aminoacyl-tRNA 30S ribosomal protein	0.06	(Stubbings et al., 2004)
Ciprofloxacin (CIP)	Quinolone	DNA gyrase (NA)	topoisomerase II and topoisomerase IV	0.25	(Schmidt et al., 2010)
Norfloxacin (NOR)	Quinolone	DNA gyrase (NA)	topoisomerase II and topoisomerase IV	1.25	(Kaatz et al., 2005)
Rifampicin (RIF)	Ansamycin	RNA synthesis inhibitors (NA)	DNA-dependent RNA polymerase	0.016	(Stubbings et al., 2004)

MIC, minimum inhibitory concentration.

### Raman spectroscopy

The samples were prepared for Raman spectroscopy with the following steps: 1 mL of the bacterial culture sample was centrifuged at 15,928 ×g for 5 min at 4°C and the supernatant was discarded. The pellets were washed with the cold Phosphate-Buffered Saline (PBS) buffer (pH 7.4). The pellets were re-suspended in formaldehyde (4%, Sigma-Aldrich, MO, USA) and fixed for 2 h at 4°C in the dark. The samples were washed twice with the cold PBS buffer. Then, a 2 μL drop was spotted on an aluminum coated slide (LiMedlon Gmbh, Mannheim, Germany) and dried in air at room temperature. Single cell Raman spectra (SCRS) were obtained with a Confocal Raman Imaging System (Nanobase, Seoul, South Korea) equipped with a 532 nm DPSS laser (Leading tech, Shanghai, China), microscope body with MPLFLN 40X objective (Olympus, Tokyo, Japan), spectrometer (Nanobase, Seoul, South Korea), and charge-coupled device (Atik cameras, Bawburgh, UK). The spectrometer grating was 1,800 gr/mm. The laser power used on the sample was 2 mW. The total acquisition time for each spectrum was 25 s. Twenty single-cell Raman spectra were collected from the samples treated with antibiotics. The SCRS were analyzed in the 400–1800 cm^-1^ region, and processed using R software version 3.6.2 using the Chemospec package (Hanson, 2015). The function “baselineSpectra” with method “als” was used to correct the spectrum baseline using 2nd derivative constrained weighted regression. Normalization of the spectra was performed using the function “normSpectra” with the method “Totlnt”. The “Totlnt” is a method of normalizing each y-value by dividing it by the sum of the y-values of a given spectrum.

### Flow cytometry

1 mL of the cell suspension was collected by centrifugation (15,928 ×g, 5 min, 4°C). The pellet was resuspended in 1 mL of sterile PBS buffer (4°C). Each sample (100 μL) was diluted with 900 μL of sterile PBS buffer in a 1.5 mL amber colored microcentrifuge tube. Bacterial viability was assessed using the LIVE/DEAD^TM^ BacLight^TM^ bacterial viability kit (Invitrogen, MA, USA) as per the manufacturer’s instructions. 987 μL of sterile PBS buffer was added to 10 μL of the prepared bacteria sample. These samples were immediately stained with 3 μL of a mixture of SYTO 9 (5 mM final concentration) and PI (30 mM final concentration) and incubated at room temperature for 15 min protected from light. All samples were measured using a CytoFLEX flow cytometer (Beckman coulter, CA, USA). The acquisition settings were as follows: intensity threshold for FSC channel, 10,000; gain value for FSC, 150; SSC, 60; FITC, 50; and PE, 20. Events were collected for 1 min at a flow rate of 10 μL min^-1^. The green (fluorescein isothiocyanate [FITC], 525/40 nm) and red (phycoerythrin [PE], 585/42 nm) fluorescence and forward (FSC) and side (SSC) optical scattering were recorded. Two biological repeats and three technical repeats were performed for each sample. The bacterial cells were analyzed for FSC, SSC, and fluorescein isothiocyanate (FITC) channel to observe changes in cell size, complexity, and nucleic acid composition following antibiotic treatment, respectively. The distinct bacterial populations (live and dead cells) were gated based on the different viability stages in density plots using the R package “alphahull”. The events in the gated as a live cell were extracted and converted into four variables (FSC, SSC, FITC, and PE) using a standardized range of 0 to 1. The FSC and FITC variables reflected cell sizes and nucleic acid content, respectively, and there were plotted on a scatter plot. The scatter plots were divided into 10 x 10 bins. The events in each bin were counted and used to calculate the phenotypic diversity. The Shannon’s diversity index (alpha diversity) and beta diversity were calculated using the R package “vegan”.

### Statistical analysis

Discriminant analysis of principal components (DAPC) was used for clustering SCRS according to antibiotic treatments. DAPC was performed by using the “dapc” function in the R package “adegenet”, which first transforms the data using PCA and then performs a discriminant analysis on the retained principal component. The SCRS was converted using PCA by the “dudi.pca” function in the R package “ade4”. The clusters were then identified using discriminant analysis (DA) using the “lda” function in the R package “MASS”. (Jombart et al., 2010). The distances between clusters in the DAPC plots were expressed by computing the average distance between each centroid of the control and each antibiotic-treated group. A two-sample t-test was performed based on the results of a normality test with the Shapiro-Wilk test to calculate the significance of the difference in the ratio of live/dead cells between the control group and the antibiotic-treated sample. In addition, the significance of the difference in SCRS between the control group and the antibiotic-treated sample was calculated. Raman peaks with a significance of p-value < 0.01 were selected and visualized with a heatmap. The significance of the difference in the phenotypic alpha diversity (D_a_) between control and antibiotic treated samples was investigated using a two sample t-test. The phenotypic beta diversity (D_b_) was visualized using a Non-metric Multi-dimensional Scaling (NMDS) plot with Bray–Curtis dissimilarity distances using the R package “vegan”. The analysis of similarity (ANOSIM) was completed using the function “anosim” within the R package “vegan” to calculate the significant difference between clusters in NMDS plot.

## Results and discussion

### Bacterial viability under antibiotic treatment

**Figure 1:**
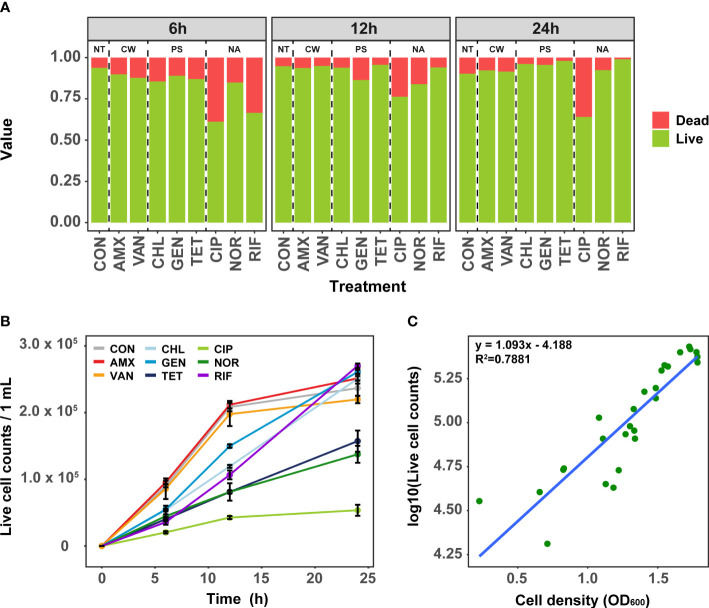
Analysis of cell viability in antibiotic-treated samples using Flow cytometry. **(A)** The change of ratio of live and dead cell over time. **(B)** Change of the live cell counts in samples treated with antibiotics over time. Error bars represent the mean ± SD. **(C)** The correlation between OD _600_ and bacterial live cell counts for each samples. NT, antibiotic non-treatment; CON, control.

To elucidate the effect of antibiotics treatment at MIC on the growth of *S. aureus*, we first quantified the time-series of cell viability using flow cytometry. The viability varied slightly depending on individual antibiotics rather than the mode of action of antibiotics. The dead cell ratios ranged from 6.2% to 38.1% (mean: 16.8%) at 6 h (**Figure 1A**). When treated with CIP and RIF, the dead cell ratio was 38.1% and 31.6%, respectively, indicating a more marked effect on cell viability compared to other antibiotics. Cell viability was mostly recovered after 24 h under all antibiotic treatment, where the proportion of dead cells decreased on average from 16.8% to 8.7%. Although the dead cells treated with CIP and AMX decreased slightly compared to 6 h, it was observed that the number of dead cells in other antibiotic-treated samples decreased significantly after 24 h of antibiotic-treated (t-test, P<0.01). Since the effect of antibiotics is closely related to the fitness of bacteria, the effect of antibiotics may have been reduced in highly nutritious media such as TSB (Steixner et al., 2021). Taken together, these results suggest that the treatment of antibiotics at MIC results in the adaptation of *S. aureus* within 24 h.

Analysis of growth curves as a simple phenotypic test using live cell counts revealed that the initial growth rates at 6 h and 12 h were clearly different depending on the mode of action of antibiotics (**Figure 1B**). In general, antibiotics related to cell wall synthesis inhibition had little growth inhibition compared to the control, whereas significant inhibition of growth rate was seen for antibiotics with protein synthesis and nucleic acid inhibitory activity. Although the cells treated with TET, CIP, and NOR did not recover to their growth maximum even after 24 h, the cells treated with TET and NOR showed better recovery than CIP. These results are consistent with the data obtained by measuring the OD_600_ (**Figure 1C**). Despite cultures being grown in separate culture and treated with different antibiotics, both the cell viability and growth curves showed similar results among these samples.

### Convergence of phenotypic diversity

**Figure 2:**
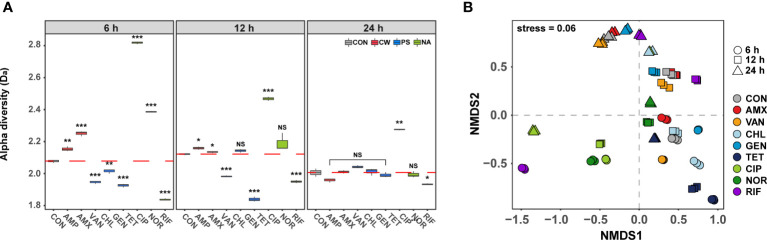
Differences in phenotype diversity between antibiotic treated samples over time by FSC-FITC data from flow cytometry. **(A)** Box plots depict alpha diversities (D_a_) of live cell distribution by Shannon index. The red dashed line means the average of control. The difference was considered significant at a P-value (NS, P > 0.05; *P < 0.05; **P < 0.01; ***P < 0.001, *t*-test). **(B)** The phenotypic beta diversity (D_b_) analysis of the flow cytometry data separating samples according to the antibiotic treatment by non-metric multidimensional scaling (NMDS) based on the Bray-Curtis distance metric.

To further characterize the phenotypic changes under antibiotic treatment, we compared the phenotypic diversity between control and treated samples using FCM. In the initial stages (6 h) of antibiotic exposure, a unique difference in the alpha diversity (D_a_) was observed according to the modes of action (t-test, P< 0.001, **Figure 2A**). The D_a_ increased when antibiotics which inhibit cell wall synthesis and DNA gyrase were used, but decreased when antibiotics which inhibit protein and RNA synthesis were used. It was confirmed that significant changes in cell size and nucleic acid content were detected depending on the mechanism of action of the antibiotic even after short-term treatment at the sub-lethal concentration (Walberg et al., 1997). The difference in phenotypic diversity according to the modes of action of these antibiotics could be a result of differential gene expression of specific resistance mechanism related genes (Hanson, 2015; Knudsen et al., 2016). Regardless of antibiotic class, the difference in the D_a_ decreased gradually over time compared to the control, and there was no significant difference at 24 h except for samples treated with CIP, which inhibits the bacterial cell growth and samples treated with CIP showed the lowest viability (**Figure 1A**). These results indicated that even though the different mode of actions influenced the D_a_ differently, the D_a_ converged into almost identical adapted states with similar orbits of phenotypes if the concentration of antibiotics was adapted. These results are consistent with the similar genotype and phenotypes of bacteria that have shown adaptation in laboratory adaptive evolution experiments (Hanson, 2015; Jahn et al., 2017). Although the short-term cultivation did not produce sufficient genetic evolution, the temporal changes of gene expression due to the abiotic stress returns to a new steady-state level close to that of unstressed cells, leading to the congruence of phenotypes (Lopez-Maury et al., 2008). From this point of view, although the D_a_ of samples treated with CIP was still significantly different from the control at 24 h, considering the decreasing trend, the diversity difference from the control group is expected to disappear as the cultivation period is increased.

The phenotypic beta diversity (D_b_) also demonstrated similar changes as seen for the D_a_ in the phenotype of subpopulations of *S. aureus* treated with the nine antibiotics (**Figure 2B**). Analyses of similarities (ANOSIM) were used to test for significant differences between samples according to the modes of action of antibiotics over time. The D_b_ of antibiotic-treated samples showed significant distance from the control after 6 h (ANOSIM, *R* = 0.81, P < 0.01), but most of those formed a tight cluster with the control after 24 h (ANOSIM, *R* = 0.36, P < 0.01). The phenotypic changes over time were found to exhibit high similarity under different antibiotics, although samples showed non-monotonic phenotypic changes even after 24 h. The observed phenotypic convergence to similar orbits clearly suggested that there is a phenotypic direction in which their fitness increases in microbial adaptation to antibiotics.

### Convergence of single cell phenotypes

**Figure 3:**
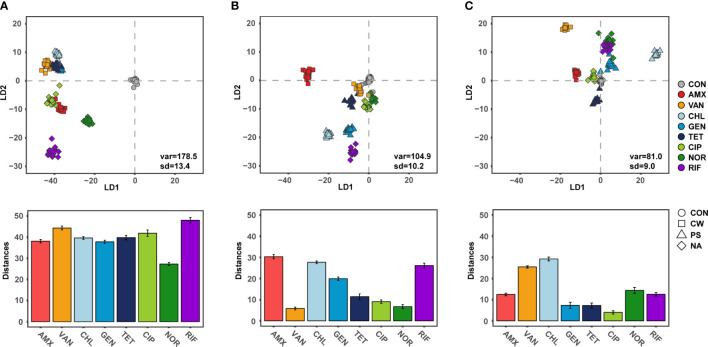
Discriminant of *S.aureus* according to the antibiotic treatments and Distance from centroids of control group to centroids of each groups. **(A)** 6 h treatment, **(B)** 12 h treatment, **(C)** 24 h treatment.

Raman spectroscopy can measure the single cell biomolecule composition (Hong et al., 2021), and thus determine the response of the phenotypic change to exposure to antibiotics (Athamneh et al., 2014). The SCRS results formed a tight cluster for each individual antibiotic at 6 h, suggesting that Raman spectroscopy is suitable for high-resolution analysis of phenotypes adapted to antibiotic exposure. These SCRS results were remarkably farther apart from the control as observed in a DA-PC plot (**Figure 3A**). These results were consistent with the fact that phenotypic diversity differed significantly from the control in the early stage of antibiotic treatment. Raman spectroscopy is a powerful approach for comparing the phenotypic difference of bacteria according to the treatment of various antibiotics with high resolution, and comparative research was also conducted at sublethal concentration of antibiotic treatment (López-Díez et al., 2005; Athamneh et al., 2014). Since ceftazidime, a cell wall inhibitor, inhibits the expression of *acrAB* encoding a multi-drug efflux pump, bacteria treated with ceftazidime were found to have decreased Raman peaks related to lipids and proteins (Athamneh et al., 2014; Peng et al., 2019). And CIP, a DNA gyrase inhibitor, inhibits the expression of *gyrAB* encoding DNA gyrase, thereby reducing nucleic acid related peaks in the bacterial SCRS (Athamneh et al., 2014; Germond et al., 2018). As is consistent with these previous studies, bacterial cellular biochemical biomolecules were clearly distinguished according to the defense mechanism against antibiotics, which indicates the effect of antibiotics on the bacteria continues for 6 h after treatment, even at sub-lethal concentrations.

The distance between SCRS clusters with antibiotic treated cells, which differed from the control cells, gradually decreased as the cultivation periods increased (**Figures 3B, C**). The SCRS analysis showed a mean distance of 39.6 (± 5.8) for cultures after 6 h of cultivation and this was significantly decreased to 17.1 (± 9.5) and 14.1 (± 8.4) after12 h and 24 h, respectively (t-test, P < 0.05). These results indicate that cellular biochemical biomolecules progress in similar orbits through bacterial adaptation in response to antibiotic exposure within short-term periods, regardless of the type of antibiotic. In laboratory evolution experiments, the phenomenon of phenotypes or genotypes convergence in the process of adaptive evolution against abiotic stress for long generation is not a rare phenomenon (Horinouchi et al., 2015; Imamovic et al., 2018). While phenotypic convergence for long generation is decided by genetic evolutionary constraints in the adaptive evolution dynamics, other factors may be more important in the convergence of phenotypes in the short term by gene expression rather than genetic evolution. One explanation of these results is that there is selective pressure from exposure to antibiotics which gives rise to a sub-population of identical cells. In these results, phenotypic diversity or deviation from normal cellular biomolecules increased during the earlier stages of exposure to antibiotics which act as cell wall synthesis, DNA gyrase or RNA synthesis inhibitors, and then decreased as cultivation periods increased. Among single cells with various phenotypes due to subtle differences in gene expression, the phenotype can converge where changes offering a fitness benefit will be dominant. Divergence in fitness is a key strategy to drug resistance in bacteria (Melnyk et al., 2015). Although there is still a lack of research on divergence in fitness at the single cell level, these results suggested that phenotypic divergence contributes quickly to adaptation from antibiotics. Another possibility is that the role of antibiotics at sub-lethal concentrations changed from antibacterial agents to signaling molecule *via* bacterial memory, leading to a similar phenotype. Antibiotics at sublethal concentrations can serve as signaling molecules and cause alterations in biofilm formation, quorum sensing, and gene expression (Andersson and Hughes, 2014). When treated with protein synthesis inhibitor antibiotics, all three samples were initially clustered similarly with low phenotypic diversity. The convergence of phenotypes, while maintaining the similarity of phenotypes between samples along with the recovery of phenotypic diversity, could result in the reduced antibacterial effect of the antibiotics. Several studies demonstrated that in the case of similar constraints, due to a limited number of adaptations which are possible in response to a specific selection pressure, selection for similar traits seems to have led to similar responses (Losos, 2011; Germond et al., 2018).

In addition, signals detected when exposed to specific stimuli can trigger short-term memory or learning behaviors in which bacteria form memories for the stimuli and respond more rapidly or more broadly to the signals on subsequent exposures (Germond et al., 2018; Bhagirath et al., 2019). These responses are regulated through the expression intensity of genes without changes to the DNA sequence, and in genetic expression, these bacterial memories might contribute to the adaptation of antibiotics and should be considered in future studies.

### Phenotypic changes in cellular biomolecules

**Figure 4:**
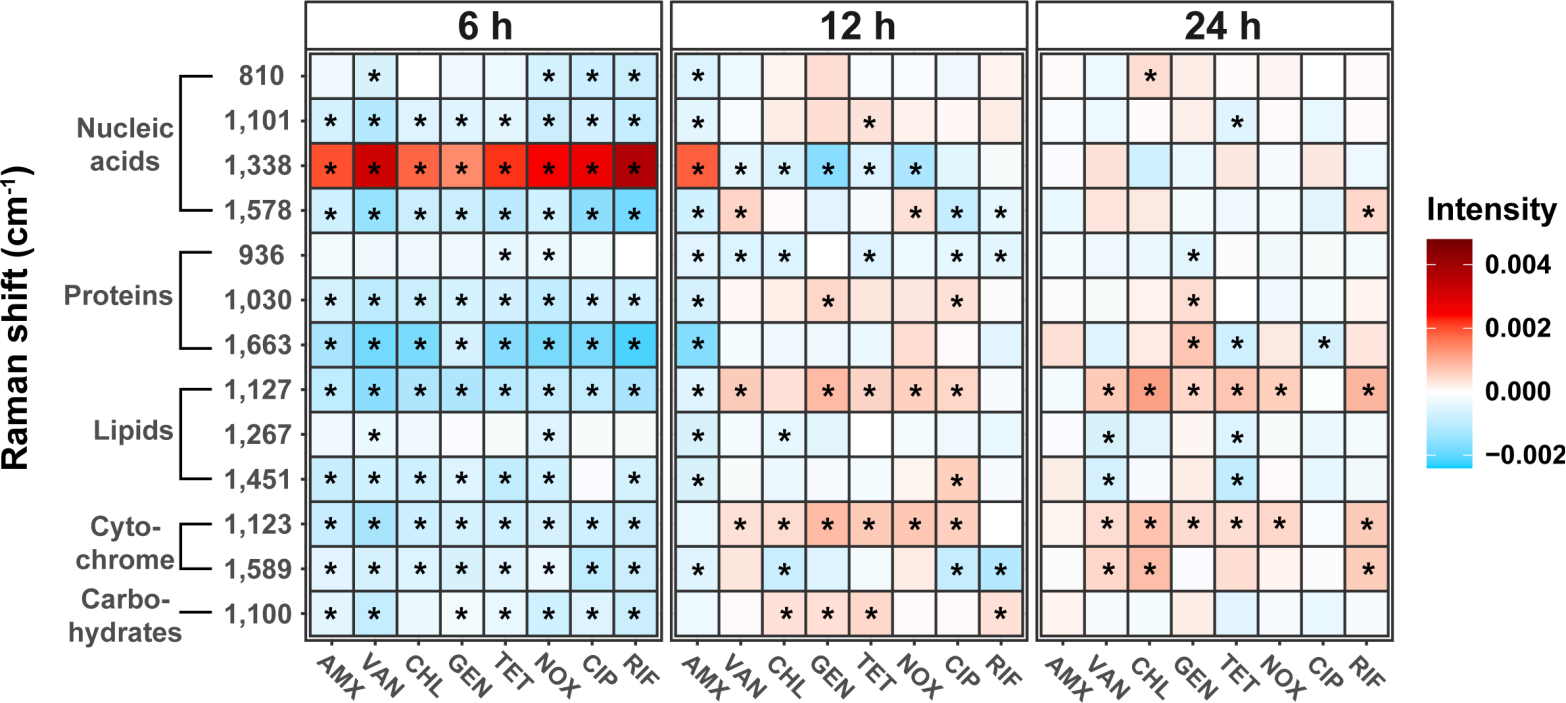
Changes in bacterial biochemical composition with different mechanisms of antibiotics and treatment time using Raman spectrum. Heat maps were generated to show the peak intensity trends over time in antibiotic treatment. Each antibiotic-treated sample’s mean spectral intensity was colored by subtracting the average spectral intensity of each time control group. The difference from control was considered significant in P-value (*P < 0.01, *t*-test).

Heat maps of the Raman intensities showed noticeable intensity changes for the major nucleic acids, proteins, lipids, and cytochrome Raman bands (**Figure 4**). The mean value of the spectral intensity of each antibiotic treated sample was colored by subtracting the average value of the spectral intensity of each time control group. Assignment of the Raman peaks was notated in **Table 2**. Regardless of the class of antibiotics, the antibiotic treatments in *S. aureus* at 6 h significantly changed the Raman intensities of cellular biomolecules including lipids, proteins, nucleic acids, cytochromes, and carbohydrates (t-test, P-value < 0.01). It is worth noting that the peaks attributing to the nucleic acids located at 1,338 cm^-1^ increased remarkably, whereas most Raman intensities were reduced by antibiotic stress after 6 h cultivation. Most of the nucleic acid related peaks were found to decrease with growth inhibition by antibiotic treatments. An exceptional increase at 1,338 cm^-1^ during the Raman peak associated with DNA can be explained by DNA damage (Sofińska et al., 2020). The SCRS under antibiotic treatments reveals a tendency for bacterial cells to decrease protein and lipid content when exposed to abiotic stress and this is enough to hinder growth (Teng et al., 2016; Germond et al., 2018).

**Table 2:** The Raman frequency of antibiotic-treated samples with significantly different intensities compared to antibiotic non-treated samples.

Raman shift (cm^-1^)	Assignment	Group	Reference
810	C-O-P-O-C in RNA backbone	Nucleic acids	(Schuster et al., 2000)
936	C–O–C linkage, C–C stretch., α-helix	Carbohydrate, protein	(De Gelder et al., 2007)
1,030	δ(CH) bend., Tyr, Phe	Aromatic compound	(Schuster et al., 2000)
1,100	mainly -C-C-(skeletal), C-O, def(C-O-H)	Carbohydrates	(Schuster et al., 2000)
1,101	Symmetric phosphate stretch. (DNA)	Nucleic acid	(Teng et al., 2016)
1,123	CH Phe	Cytochrome	(Notingher and Hench, 2006)
1,127	=C-C= (unsaturated fatty acids in lipids)	Lipids	(Huang et al., 2010)
1,209	C–C_6_H_5_ stretch., Phe, Trp	Protein	(Notingher and Hench, 2006)
1,267	Lipids	Lipids	(Van Manen et al., 2005)
1,338	Adenine, guanine, tryrosine, tryptophan	Nucleic acids	(Strola et al., 2014)
1,451	C-H_2_ def, Lipids	Lipids	(Notingher and Hench, 2006)
1,578	Adenine, cytosine, guanine	Nucleic acid	(Maquelin et al., 2002)
1,589	ν(C−C)	Cyt c.	(Cui et al., 2018)
1,665	Amide I	Amide I	(Maquelin et al., 2002)

In addition, large variability related to nucleic acid content could also be identified in the SCRS. Antibiotics resulted in changes to the various nucleic acid processes in bacterial cells, such as DNA fragmentation and gene expression for defense and repair (Moritz et al., 2010). Using Raman spectroscopy, we were able to distinguish changes in cellular biomolecules according to the modes of action of antibiotics, and confirmed common phenotypic changes that occur due to sublethal antibiotic treatment.

As the cultivation periods reached 24 h, most of the differences seen in the Raman intensities of biomolecules between control and treated samples were no longer observed. In particular, the Raman intensities associated with nucleic acids, proteins, and carbohydrates reverted back to levels similar to those of the control after 24 h cultivation, while cytochromes (1,123 cm^-1^) and lipids (1,127 cm^-1^) increased significantly in most of antibiotic treated samples except for AMX and CIP (t-test, P-value < 0.01). In particular, the bacterial cell membrane is essential in bacteria and serves as a significant barrier to preventing harmful chemicals from entering the cell (Willdigg and Helmann, 2021). Bacteria induce a cell envelope stress response to resist antibiotics, which are regulatory pathways that detects threats and cause protective reactions, including modifications of the lipopolysaccharides, lipoteichoic acids, peptidoglycan (Macdermott-Opeskin et al., 2022). Changes in membrane composition has been previously reported as one of the most important adaptive mechanisms in bacteria when exposed to toxic compounds such as antibiotics (Ma et al., 2021). Antibiotic resistance can be induced by this lipid-mediated mechanism, and the increase of the lipid peak in clusters at 24 h is presumed to be caused by an increase in fatty acids in the cell membrane of the bacteria or a change in the composition of membrane lipids. Cytochromes are known to be required for biofilm formation and extracellular matrix production that can resist antibiotics and other external stressors. Bacteria can cope with oxidative or antibiotic-induced stress by increasing respiration through cytochromes and also enhance susceptibility to antibiotics by decreasing outer membrane permeability. (Beebout et al., 2021). Thus, the increase in lipids and cytochromes in most SCRS can be considered a phenotypic indicator that the bacterial tolerance to antibiotic-induced stress is improved. Although it is difficult to determine that *Staphylococcus* has evolved adaptation to antibiotic resistance over generations in this study, it is worth considering the possibility that repeated adaptation through antibiotic treatment at such sub-lethal concentrations will change the antibiotic susceptibility. Further research on how the repeated exposure to antibiotic leads to changes in antibiotic resistance and phenotype needs to be considered.

## Conclusion

In this study, analyzed phenotypic changes of *S. aureus* cells that occurred during adaptation in response to antibiotic treatment at sub-lethal concentration using FCM and Raman spectroscopy. The similar phenotypic changes among independent cultures treated with different antibiotic classes, with different modes of action, indicate the existence of an adaptive direction in phenotype in short-term cultivation periods. These results provide a rapid transition to phenotypes in which fitness increases in the adaptation process, even if the initial response strategies to abiotic stress is different. This study also highlight the feasibility of phenotypic studies in long term antibiotic treatment or when investigating new antibiotic classes.

## Data availability statement

The data presented in the study are deposited in the FlowRepository, accession number FR-FCM-Z56A.

## Author contributions

GN and TK contributed to the conception of this study and designed the experiments. GN, ES, and J-KH conducted the experiments. GN, JK, and SB carried out the data analysis. GN and ES contributed to the interpretation of data. GN wrote the manuscript. TK supervised the project and provided critical comments. All authors contributed to the article and approved the submitted version.

## Funding

This work was supported by the National Research Foundation of Korea (NRF) grant funded by the Korea government (MSIT) (No.2019R1A4A1024764). This work was supported by the National Research Foundation of Korea (NRF) grant funded by the Korea government (MSIT) (No. 2020R1C1C100624912).

## Conflict of interest

The authors declare that the research was conducted in the absence of any commercial or financial relationships that could be construed as a potential conflict of interest.

## Publisher’s note

All claims expressed in this article are solely those of the authors and do not necessarily represent those of their affiliated organizations, or those of the publisher, the editors and the reviewers. Any product that may be evaluated in this article, or claim that may be made by its manufacturer, is not guaranteed or endorsed by the publisher.
